# Phenomenology, *Pokémon Go*, and Other Augmented Reality Games: A Study of a Life Among Digital Objects

**DOI:** 10.1007/s10746-017-9450-8

**Published:** 2017-11-07

**Authors:** Nicola Liberati

**Affiliations:** Department of Philosophy, University of Twente, Enschede, The Netherlands

**Keywords:** Phenomenology, Postphenomenology, Augmented reality, *Pokémon Go*, Paramount reality, Finite provinces of meaning

## Abstract

The aim of this paper is to analyse the effects on the everyday world of actual Augmented Reality games which introduce digital objects in our surroundings from a phenomenological point of view. Augmented Reality is a new technology aiming to merge digital and real objects, and it is becoming pervasively used thanks to the application for mobile devices *Pokémon Go* by *Niantic*. We will study this game and other similar applications to shed light on their possible effects on our lives and on our everyday world from a phenomenological perspective. In the first part, we will show how these digital objects are visualised as merged in the surroundings. We will point out that even if they are visualised as part of the everyday world, they are not part of it because they are still related to the fictional world generated by the game. In the second part, we will show how the existence of these objects in their fictional world has effects on the everyday world where everybody lives. The goal of Augmented Reality is not reached yet because these objects are not part of the everyday world, but it already makes our lives embedded with digital elements. We will show if these new objects have effects on our world and on how we live our lives.

## Introduction

Our world is literally saturated by digital technologies. These devices touch almost every aspects of our lives by being deeply intertwined in our everyday praxes. They accompany us by providing information, by storing our daily schedule, by keeping each other in touch, and, generally speaking, by being constantly active around us.^1^

This intertwinement between everyday activities and digital elements is becoming even more pervasive and our world is becoming inhabited by digital entities generated by computer devices like in case of Augmented Reality (AR).^2^ This technology was introduced in the 90s by an engineer of *Boeing* (Caudell and Mizell 2002; Caudell 1995; Billinghurst et al. 2015), and now, in 2017, it is used by millions of people around the world thanks to the application *Pokémon Go* developed by *Niantic*.^3^ With Augmented Reality, it is possible to intertwine digital objects with the ones in our surroundings, and, thanks to the Pokémon’s application, this “mixture” is becoming pervasively used by many users.

There are works analysing Augmented Reality in relation to legal issues (Wassom and Bishop 2014), works analysing the impact of Augmented Reality in Art (Geroimenko 2014; Aceti et al. 2013), works focussing on the emergence of this new medium from a philosophical perspective (Engberg and Bolter 2014; Bolter et al. 2013; Manovich 2006; Schraffenberger et al. 2014; Schraffenberger and Manovich 2013; Shilkrot et al. 2014), but there are very few works related to a phenomenological analysis on these new augmented objects introduced in our environment (Wellner 2013, 2015; Liberati 2013).

A phenomenological study on these augmented objects allows us to understand how they are intertwined with common objects in our everyday world from the users’ perceptual point of view. These objects are not only interesting because they are generated through new devices. They are perceptual objects which becomes part of the experience of the users. Thus, instead of focusing on the novelty of the media involved in the process, we will study how they are intertwined within the users’ life by highlighting how they are perceived by them and how their existence is founded on the overlap of different worlds. We want to understand how this intertwinement is achieved and the effects it yields on our lives from a perceptual point of view.

This paper will be divided into two main sections. The first one will focus on the intertwinement between digital and common everyday objects. In this section, we will take into consideration four AR applications in order to have actual games to work with, and we will study how these objects are perceived by the users thanks to Husserl’s and Schütz’s phenomenology. The second section will focus on the effects of these objects in the paramount reality. Our aim is to show how these augmented objects are able to modify the paramount reality of the subjects even if they are fictional objects enclosed in a finite province of meaning according to a phenomenological point of view.

## AR Games and the Intertwinement Between Digital and Everyday Objects

### AR Games

The aim of Augmented Reality is to provide the intertwinement between digital and everyday objects. The goal is to make our everyday world inhabited by digital objects and so to move the intertwinement between digital elements and users’ everyday lives to a completely different level. This kind of mix can be achieved in many ways, and they can produce very different “Mixed Realities”.^4^

We will focus our attention on four Augmented Reality applications which achieve such an intertwinement in a similar way and which are games designed for mobile devices in order to restrict our analysis. We will take into consideration the following games:

1. *Geocaching* 5
2. *iButterfly* by *Dentsu Inc.* 6
3. *Ingress* by *Niantic* and *Google*.^7^
4. *Pokémon Go* by *Niantic* 8

These games are well connected to each other, and they allow us to look at the gradual introduction of new elements in Augmented Reality. The application *Pokémon Go* uses elements coming from previous games, and so, by showing its “predecessors,” we will also highlight some of its founding elements, and we will gradually introduce the novelties generated by Augmented Reality.

#### *Geocaching*

The game *Geocaching* 9 is basically a treasure-hunt where players^10^ have to use *Global Positioning System* (GPS) location service to locate the objects of the game through devices which show their position on a map.^11^

The idea of this game is to hide “real”^12^ objects in the world and to make them “findable” only for the players who can access their GPS locations. Physical objects are located by players in “real” locations in the world^13^ and their GPS position is saved in a database. This database is accessible by players who can use the GPS data to locate the objects which are hidden in the surroundings like under a rock or in a bush. Thus, the players need the GPS locations because without knowing the position of these hidden objects, it would be almost impossible to find them.

In the application developed for mobile devices, the players can see a map of the real world where it is shown their position and the positions of the hidden objects thanks to placeholders. Players can move in the real world and get close to these objects by following the direction displayed on the map. Once the players get close enough to the object, they turn the device off and they start the search in the surroundings.

The game divides peoples in two main groups: the ones who cannot see the hidden objects of the game and the ones who can see them. The subjects who do not have the knowledge or capabilities to perceive the hidden objects of *Geocaching* are labelled as “muggle,” using a terminology coming directly from *Harry Potter*.^14^ This group of people does not have “digital” capabilities to find the hidden objects and so, like in the fictional world of *Harry Potter*, they live in a deprived world where all the *geocaching* elements are “invisible” for them. They live in the world without knowing the real richness underlying it. Differently, the geocachers, who are the people playing at this game, see this hidden world within the everyday world lived by the “muggles” thanks to the access to this database of locations. What is a bunch for a “muggle” is a “bunch hiding an object” for the geocacher.

The intertwinement between digital and real objects is made thanks to the creation of a digital database containing the positions of the real objects and its availability to players through to mobile devices.

#### *iButterfly*

*iButterfly* was developed in December 2009 by *Dentsu*. In this game, the players do not catch physical real objects thanks to the digital information stored in a database, but the players catch digital objects.

Digital flying butterflies are generated and superimposed on the images captured by the camera of mobile devices as if they were flying in the surroundings. Players, looking through the camera of their devices, see these butterflies flying all around them. They can catch these digital butterflies by moving the smartphone as if it were a butterfly net. Once captured, the butterflies can be collected and sent as gift to other players.^15^

In this case, we have a different way of intertwining digital and real elements. In *Geocaching*, real objects were made traceable thanks to digital technologies, now the application produces digital objects directly instead of using physical ones. Moreover, the objects are not placed on a map as placeholders showing their location, but they are visualised as part of the surroundings thanks to the superimposition of their images. The players, by looking through the device, see the digital butterflies moving around in the surroundings as if they were physically in the real world.

#### *Ingress*

*Ingress* was developed by *Niantic* and *Google* in December 2013. In this game, two opposite factions try to defeat each other by activating portals around the world. Portals are located in famous places of cultural significance and geolocalised with their GPS coordinates. The players have to physically go there in order to interact with these digital objects.

The places are real, but the objects placed in them are digital. The application allows the players to see a map of the real world where their positions and the ones of the digital objects are visualised as placeholders. Like in the case of *Geocaching*, by moving in the real world, the players can get close enough to these objects, but this time, in order to interact with them, the players need to use the mobile devices because the objects are generated by the digital system, and they are visible only through the devices.

In this case, we have the two kinds of intertwinement we showed in the previous applications. The objects are stored in a digital database accessible by the players and so they are made “visible” thanks to this digital access like in the case of *Geocaching*. The objects of the game are digital objects and not real ones like in the case of *iButterfly*. However, at the same time, they are not perceived as merged with the surroundings because the players see them only on the map as placeholders like in *Geocaching*.

#### *Pokémon Go*

The application *Pokémon Go* was developed by *Niantic* in 2016. It is the most pervasive Augmented Reality game ever used by far.

This application makes the world inhabited by digital Pokémon monsters. They are generated by a server, and they are located in physical places in the players’ surroundings by anchoring them to GPS locations. These objects are visualised on a digital map on the device as placeholders. In order to “catch ’em all,” as the famous motto of the game suggests, the players have to physically move in the everyday world by using the map provided by the application as navigator like in the case of *Ingress* or *Geocaching*. Once they found the Pokémon they are interested in, the system allows the players to “see” these digital objects by superimposing their images on the images captured by the camera like in the case of *iButterfly*. Players have to visualise them on the screen of the device and “catch them” by throwing digital Poké Balls which are able to encapsulate the creatures.

In this case, we have the same intertwinements we found in *Ingress* because the objects have GPS locations and they are digital. However, we also have another element. These digital objects are blended into the surroundings by superimposing their images to the ones taken by the camera like in the case of *iButterfly*.

In order to study how subjects perceive these objects and how they will affect our lives, we will take into consideration Husserl’s and Schütz phenomenology. The former will help us to understand how these objects are part of our world. The latter will help in showing the effects of these objects in our everyday world.

### Augmented Reality and the Paramount Reality

#### AR Objects Among Other Everyday Objects

We usually deal with digital objects in our lives even without using Augmented Reality. For example, in the case of a film projected in a cinema, objects are shown to the subjects seated in the cinema’s chairs. The scene shows to the spectators real and digital objects generated by special effects^16^ co-acting on the screen (Liberati 2015b). However, these digital objects are not part of the everyday world, even if they are clearly perceived by the amused audience in a cinema. These objects, even if they are vivid in the film, have no place at all in the world outside the film. They are enclosed in “fictitious” worlds generated by the devices around them like the projectors and the speakers working in a cinema.

According to Husserl, subjects in such kind of condition do not perceive objects in the everyday world, but they are immersed in a second world where they suspend the common concerns about the existence of the world around.^17^ The screen of the cinema is a flat surface which opens the access to a completely new different world. Spectators do not perceive the screen as a flat thing, but they perceive an entire world in it.^18^

As Lotz clarifies Husserl uses the term “look into [*Hineinsehen*]” in order to show how the subjects do not merely see something in a picture or in a film, but they feel themselves *in* the picture or *in* the film (2007: 174).^19^

There is a kind of immersion^20^ of the subject into this second world, and the objects of the scene are perceived as fictitious and as part of this different world. The objects might be actual within the world produced by these devices, but they are not actual in the world “outside” it where the subjects usually live.^21^

There is a clear distinction between the objects in the everyday world and the ones provided by the filmic fictional world. The former are actual objects part of the everyday world of the subjects. The latter are objects confined to the fictional world of the film. The screen is a kind of “door”^22^ opening to a different second world.^23^

In the case of Augmented Reality, we do not have this kind of distinction. The objects are produced as located in the everyday world. While in the case of films the devices produce a door to a second world into which the subjects have to immerse themselves, with Augmented Reality, the subjects live in the same world without any kind of immersion into a “second” different world. For example, in the case of *Pokémon Go* the subjects move and physically go in locations in the real world in order to capture the digital creatures.

These Augmented Reality applications do not yield any immersion *in* a different world, but they add digital elements to the surroundings. The subjects do not “look-into” the devices as if they were immersing in a second different world. They are looking *through* these devices towards the surroundings. This idea of looking *through* the technology to perceive the world is tightly related to the idea of postphenomenology where the perception of the subjects is mediated by technological devices (Ihde 1990; Verbeek 2005). Technology shapes the perceptual ability of the users in the real world instead of producing a second world where the subjects are immersed (Liberati 2016).

Augmented Reality does not provide an access to a different world, but it allows the digital element to be part of the players’ surroundings. However, this merging is not enough to make the digital objects part of the everyday world of the subjects. Even if the digital objects have the everyday world as a background on which they are superimposed, they are not part of the surroundings as other objects. They are part of the game generated by the device. These Augmented Reality games still produce “digital fantasies” even if now the digital objects are visualised in the surroundings. Therefore, even if these objects are visualised exactly as the other everyday objects, they are still part of the game-like world generated by the game.

Another possible angle to analyse these fictional objects is to think of them as still within a “screen,” even if it is a different kind of “screen”. While the classic “screen” of a theatre and in the cinema has to be abandoned because now the fictional entities are able to “live” outside of specific limited places, it is possible to see the device as a tool which turns the entire world into a “screen”. By looking through the device the subjects turn their surroundings into a surface where to visualise digital objects. Therefore, instead of erasing the screen, it is possible to think of the world as a new “screen” where to live fictional experiences. Both of these analyses, even if they enframe the change in different ways, allow us to take into account these digital entities as placed in the surroundings maintaining their fictional status. The former erases the screen showing how fictional entities jump out of the screen into the everyday world. The latter shows how Augmented Reality is able to make the entire world a screen on which visualise digital entities. Both of these analysis are relevant, but we will focus our attention only on the first one because it better shows the aim of Augmented Reality to make the digital object part of the everyday world as if they were common usual objects.

People playing Augmented Reality games can define the others as “non-players” or as “muggles” because they do not “see” the richness of the world. However, this “richness” is part of the game-like world generated upon the everyday world and not the everyday world.

In order to analyse the relations among everyday world and the world generated by these games, we need to introduce Schütz’s phenomenology.^24^

#### AR Objects as Part of a Game

Digital objects generated by Augmented Realities are related to the activation of these games. These objects are application dependent. This simple fact clearly points out the problem Augmented Reality is facing. The aim of this new technology is to merge digital objects in the everyday world. However, for the applications we analysed, the digital objects are “merely” part of the world generated by that particular application which uses the everyday world as background, and so they are not part of the everyday world where subjects usually live.

Only *Geocaching* application points to real physical objects, the other applications produce digital objects which are valid only within the game. The butterflies flying all around the players are invisible to common people, and non-players do not even suspect their existence. The only thing non-players will see are the frenetic actions made by some people moving compulsively their smartphones in the air, but they will never see or suspect the existence of the digital butterflies. *Ingress* and *Pokémon Go* move in the same direction. They add digital objects like digital portals and creatures to the everyday world, but these entities are not visible, nor accessible, by the ones who do not use the devices and who do not play the same game. Do they have any effect on the everyday world even if they are not accessible by everybody? Are these entities different from objects introduced by other games?

This kind of co-existence among realities is well analysed by Schütz’s phenomenology which identifies a paramount reality^25^ where people usually live in their everyday lives and different finite provinces of meaning (Ayaß 2016; Waltemathe 2014) generated by dreams, arts, literature, and games (Tuckett 2015: 74). In these worlds, there are fictional objects which are experienced by players or dreamers, but which do not have any relevance outside of the limited world generated by the special activity.

According to Schütz, there is one paramount reality on which finite provinces of meaning are built (Steets 2014). The paramount reality is founded on many different elements (Schütz 1962: 342). However, without going into details, we can just point out this special reality is founded on the work [*Wirk*] made by the subjects (Bischur 2014; Barber 2015), on the attention to life [*Attention à la vie*] the subjects have (Schütz 1962: 226), and on the fact that this paramount reality is the ground where everybody has to live.

The other worlds are called finite provinces of meaning which are “merely” valid within their limits like in the case of films. These worlds are not “inferior” or less important for the subjects, but they are built upon this paramount reality, and they do not “gear” (Schütz 1962) in the same way of the paramount reality.

These finite provinces of meaning could be so overwhelming sometimes subjects are not even aware of being part of it like in the case of dreams. Subjects can live in it as if it were the paramount reality, and only after, when they awake, recognise the finiteness of the world just experienced. They could not even have control of what is happening in some provinces as nightmares clearly show (Schütz 1962: 241). Other provinces allow subjects to be more “aware” of them as in the case of the world generated by literature and arts. Thanks to a technology like a book or a film, an entrance for a different world is created where the subjects consciously enter or, using a Husserlian terminology, “look-in”. For example, in films, subjects simply “live within” (Psathas 1998: 222) the “filmic reality” as a second world build upon the everyday one (Liberati 2015b).^26^

These worlds are “personal” finite provinces of meaning where there are no interactions with other subjects coming from the paramount reality. The subject can dream of meeting another person, but this person is not a person in the everyday world. In the filmic reality and in the world presented by a book, subjects do not meet real people, but representations or characters of the plot who have nothing to do with the paramount reality (Casebier 1991).^27^

In the case of games, we have fictional worlds too (Tuckett 2015: 74), but they are not “private”. The finite provinces of meaning generated by the games are open to other subjects, and, especially in our case, the games are founded on the co-actions of different players. The objects in these worlds are not present for one subject only, but they are present for an entire community. For example, in the case of games like chess, the pieces on the board are objects for the two players and for the ones who are watching the match. They are not “private” because they are co-lived by many subjects at the same time.

Some games have clear spatial limits in the paramount reality. The chessboard is the place where the game of chess and its objects are meaningful. For example, the pieces of chess are meaningful only within the chessboard where they can be moved and where they can interact with other pieces, but they lose their meaning outside of it. In the same way, the action of moving the pieces has a meaning only within the board. The entire community watching the game see the the players acting according to the rules dictated by the game (Goffman 1974: 249). The people who are not involved in the match see merely the movement of hands and the movement of insignificant physical objects, while the people enjoying the game see the action of moving a piece on the board (Fine 2002: 183–185)^28^ Therefore, in some cases, the provinces of meaning are clearly limited to certain places, and the players cannot exit it.^29^

There are other games which do not have spatial limits. For example, the playful world of the game of children where they interact with others while co-acting with illusionary entities clearly shows how there are no spatial limits besides the ones posed by the players. The entire world is a potential playground without any limit. Houses, parks, streets, and dangerous places can all become the field where the fantasy world is located. Thus, the fictional objects generated in the game are not confined in a specific place because they are free to be located everywhere in the paramount reality. The actions of the children and the objects generated by the game are still meaningful only within the world of the game, but they can be performed *everywhere* because this fictional world can be played *everywhere*.

In the case of the Augmented Reality games, we showed the digital objects cannot be restricted to a confined area because they are free to be visualised everywhere in the world. Even if these objects are clearly limited to the finite provinces of meaning of the AR games, they are everywhere.

Augmented Reality allows players to visualise digital objects in the surroundings without any spatial limit. However, these objects, even if they can be located everywhere, are still restricted to the world of the game generated by the Augmented Reality. It is a game without any spatial limit, but it is a finite province of meaning.

For a technology which aims to merge digital objects in the everyday world, the restriction of its objects to finite provinces of meaning cannot be enough.^30^ Even if they achieve the intertwinement between digital and real objects because they managed to visualise objects located in the everyday world, they generate “mere” fictional entities which are restricted to their finite province of meaning. The intertwinement is achieved but in a game-like world. Thus, the original goal of producing digital objects in the everyday world is not achieved yet, but this limitation does not preclude these objects to have effects on the paramount reality.

## AR Effects on the Paramount Reality

### Enclaves and Movements Among Different Realities

Augmented Reality, at the moment, allows intertwining digital objects with our physical world limiting these objects within the limits of finite provinces of meaning. Obviously, the existence of these objects has effects within the limits of these worlds because the entire play is founded on them. During the game, players are affected by these objects. For example, players philically go to special places of the game where Pokémon are located in order to catch them. However, these objects have effects even in the world “outside” the game. They change the paramount reality even if they are “merely” part of the finite province of meaning.

A game creates its own objects and constitutes its own world where the players live. As Goffman highlights (1961), each game has its own rules to which the players are related thanks to their sole participation (Ducharme and Fine 1994: 94). These rules define the actions the players can do within the game, and they dictate also the values of the objects in the finite province of meaning because they define the goals of the game.^31^ If the game’s goal is to catch all the Pokémon, the players see the creatures valuable and, with it, even the locations where they are used to appear acquire value. Players want to get the valuable Pokémon and so they go in the places where these Pokémon appear making the places valuable for them.^32^

This fact generates a possible interaction between realities and the values they impose to subjects. They can be the ground on which those realities influence each other.

As Schütz pointed out, the overlap of multiple provinces is simply impossible (Schutz and Wagner 1970: 256) because the world defined by one province clearly clashes with the one produced by other one (Schütz 1962). For example, while children play among fictional entities, their finite province of meaning clashes with the world of their parents where there are no such fictional objects. In our case, the world generated by the Augmented Reality game *Pokémon Go*, where Pokémon are all around the players, clearly clashes with the world outside the game where there are no such digital entities. They are different realities, and subjects cannot be in two worlds at the same time. However, even if it is impossible to live in many realities at the same time, it is possible to move from one province to another one (Chojnacki 2012).

The objects of the games are taken for granted thanks to what Schütz calls the “epoché of the natural attitude” (1962: 229). Subjects simply suspend their doubts on the existence of the objects around, and they live among them acritically. According to Schütz, subjects live constantly surrounded by many different realities and the fact they live in one of them is determined only by the suspension of doubts made by the subjects.^33^ This kind of element clearly shows the possibility of moving among provinces because they can move among these realities by changing where they want to suspend their doubts.^34^ Every time they change from one province of meaning to another one, they change abruptly the world around them. For example, in the case of games, their entire world collapses when the games end, and the objects in it vanish. Another example can be dreams. While dreaming, subjects are immersed in a finite province of meaning. When they wake up, they change the entire world where they live moving from the finite province of meaning of the dream to the paramount reality. Even if these realities cannot overlap, the subjects can move freely among them, and this element is the one producing the intertwinement.

According to this ability to move among realities, the subjects can live in “enclaves” where there is a tight intertwinement of these provinces (McDuffie 1995: 216). For example, while reading a text, the reader can stop the reading in order to have a cup of coffee. In this case, the reader can move from the finite province of meaning of the text to the paramount reality where the coffee is. This shift from the world of the text to the paramount reality does not make the province of the text inaccessible. Thus, the reader can re-enter it quite easily, even while drinking the coffee. Moreover, the reader can move constantly from the world where the reader sips the coffee to the world of the text and *vice versa*.

Even if the overlap among provinces is impossible, it is quite possible and usual to shift from one reality to another one many times per day. This constant movement among realities intertwines the activities related to these worlds together.

The objects within a finite province of meaning are clearly related to this fictional world, but they do have effects outside of it because the subjects do not live merely within one single reality. Thanks to the possibility of changing realities and moving among them, the subjects cannot be restricted to the “mere” paramount reality. They constantly leap from this world to other fictional worlds. Therefore, even if these fictional objects generated by games are clearly existent and meaningful in their finite province of meaning, they do affect the lives of the subjects because they are free to intertwine the activities of the paramount reality with the ones of the games.

According to this analysis, the game is not so well encapsulated within its finite provinces of meaning because it is possible to intertwine the activities coming from different realities together. Thus, the place and the time the game is played becomes crucial because these elements define where and when these activities can be played and so when and where they can be intertwined with the others.

Their spatial position is important. The objects of a game are clearly enclosed within the finite province of meaning of that game. However, games take place in the paramount reality because every finite province of meaning is founded on it. Therefore, even if the objects of the game are meaningful only within the game, they have a spatial position which is also a spatial position in the paramount reality. The spatial position is a link between the different realities and so it can be used by the subjects to intertwine their activities.

The time when the game is played is important too. The time of the finite province of meaning of the game is different from the time in the paramount reality. For example, players can imagine forcing years to pass like seconds in the game, but they cannot do it in the paramount reality. However, the game is still played in a time which is the time of the paramount reality where everybody has to live.^35^ Therefore, even this element can be used to build intertwinements between the activities of the game and the ones in the paramount reality because they both have a temporal position in the paramount reality.

As we showed, the Augmented Reality games we introduced do not have spatial limits because they can be played everywhere. The objects of these games have GPS locations which are actual spatial locations in the paramount reality and which are everywhere in the world. There is no spatial limit where the game is confined, and the game “wraps” completely the paramount reality. *Geocaching*, *iButterfly*, *Ingress*, and *Pokémon Go* have objects everywhere, we cannot point a finger to a single place which is “safe” from their contamination because space of the game is constituted of every spatial position in our paramount reality.

Moreover, these games do not have temporal limits too. They can be defined as infinite games (Carse 1986) because they do not have any clear temporal boundaries. They do not have a clear winner and so the game never ends.^36^ The game is always on and so subjects can freely access it anytime they want.

The game *Geocaching*, as the other games we analysed, do not have winners because the aim of the game is to find as many objects as possible. Nobody wins and nobody loses. It has no limitation in time and space because it is always possible to open the application and check for objects in the surroundings and, potentially, it never ends because players will always be able to seek for other objects.

*iButterfly*, *Ingress*,^37^ and *Pokémon Go* have the same basic characteristics. The aim is not to win but to keep the game going on by capturing new butterflies, by interacting with the other faction for the control of portals, and by catching Pokémon in the surrounding. Moreover, they do not have limitation in space and time just as the game *Geocaching*.

The world generated by these games never ends, and it has no spatial limits. Subjects can play them everywhere and at any time. Thus, the intertwinement between the actions made within the game while playing and the actions made in the paramount reality has the chance to be tightly intertwined in a never ending process and everywhere in the world.

In Nagoya (Japan) a news attesting the sighting of a rare Pokémon hiding in a famous park was read by many players. Even if the information was false and there was no rare Pokémon hiding in that place, many people went there in the middle of the night hoping to find it (Ashcraft 2016). That place was a park, but it could be anywhere. Moreover, the players were attracted in that place in the middle of the night showing that the game was always active without any temporal limit.

The absence of spatial and temporal limits for these game makes them pervasive, and it allows a constant tight intertwinement of the action within these finite provinces of meaning and the actions made in other realities like in the paramount reality.^38^

This tight intertwinement between the actions in the game and the paramount reality is well testified by the game *Ingress*. Players got arrested because they were using the mobile devices in spatial places where they should have turned the devices off like in police stations. The players were attracted there because of the existence of an important location according to the finite province of meaning generated by *Ingress*. They were in this provinces of meaning while interacting with the fictional objects, but their locations were a place where the use of digital devices was prohibited in the paramount reality. Thus, they got detained (Foster 2012).

Obviously, we do have the same kind of possible intertwinement even with games limited in space and time because their actions and objects have spatial and temporal positions in the paramount reality as well. However, we can clearly see their intertwinement are different from the ones in Augmented Reality because they are limited to where the game is located and when it is performed. In the case of Augmented Reality, the game does not have these limitations. For example, in the case of chess, the space where the game can be performed is the chessboard, and only the actions made within that space are actions in the game and, at the same time, actions in the paramount reality. However, in the case of the Augmented Reality games we described, the play is not limited to the chessboard, but it spreads all over the paramount reality.

### AR Games and Effects on the Players

The objects of these games are related to the finite provinces of meaning of their games. However, as we showed, they can have effects outside of them because the subjects can move constantly from one world to another one, and so they can intertwine the actions and praxes related to these different worlds together.

While the subjects are in the paramount reality, they can immerse themselves into the game just to come back in the paramount reality after few seconds. They can open the application on the smartphone, capture one Pokémon, and re-emerge in the paramount reality. During this movement between realities, the actions of the subjects in the paramount reality could be influenced by the digital objects which are “meaningful” only in the province of meaning of the game.

As we showed, the objects have the same spatial position of locations in the paramount reality. Moreover, the players can constantly immerse themselves in them because the games have no temporal limits.^39^ Therefore, the values the games dictate are valid only within their finite provinces of meaning, but they share with the paramount reality the same spatial locations and they are always accessible. Since the subjects are free to move among realities, it is possible the action made in the paramount reality is affected by the values of the objects in the game because they share the same places and the subjects can always easily jump in the game.

For example, players, while going to work, could not only open the application for catching one Pokémon, but they could modify the route they usually follow in order to pass by a convenient place for capturing the Pokémon. Even if the objects are “encapsulated” within the finite province of meaning of the game, they are able to change and to interact with the actions performed in the paramount reality like the action “going to work”. The special locations where to find a Pokémon are valuable only within the finite province of meaning of the game because it is the game which gives it, but the fact it is also a place in the paramount reality makes this value leak outside of that province.

Subjects are not “slave” of a single reality. They can move among many of them, and this movement hybridises the activities performed in the paramount reality with elements coming from other provinces because they can decide to act in a certain way in the paramount reality partially following the values coming from other realities. Therefore, even if these digital objects are meaningful only within the finite province of meaning where the players immerse themselves in order to play, they do have effects on the players’ actions in the paramount reality.

The effects are not limited to the actions in the paramount reality of the players, but they affect even the non-players.

### Effects on the Non-players

The paramount reality is intrinsically intersubjective, and it is defined as the world where every subject has to live. Thus, the digital objects affect the paramount reality of everybody by affecting the actions made in the paramount reality by the players.

The play is performed in the finite province of meaning of the game, but it has elements also in paramount reality. Therefore, the actions within the game are seen by the non-players too, even if they do not get their meaning because they are outside the finite province of meaning. For example, in the case of *iButterfly*, the players move their smartphone frenetically in order to catch the digital butterflies flying around them. The non-players do not see the butterflies, and they do not see the actions of these players as action for “catching” the digital objects. However, they see their motions with the smartphone in the space of the paramount reality.

In the case we proposed of a player changing the route to work in order to pass by a convenient place where to catch Pokémon, we showed how the game have effects in the actions of the paramount reality of the players. However, it has also effects on the non-players because they all are in the same paramount reality. For example, if many players gather together in the same place and the streets around that place were originally not designed to support such a massive traffic, they can provoke a traffic jam. This traffic jam directly affects the people around even if they are non-players.^40^

Even if the values dictated by the game are not valid for the non-players, the spatial location where the game is performed becomes embedded with elements deriving from those values. The valuable location for the players becomes “relevant” even for the non-players because of the power of “attraction” it has for part of the population which makes the players act in a certain way in the paramount reality. Therefore, the game has the power to re-shape the paramount reality by adding relevant elements to it thanks to fact the players live in the paramount reality too. Moreover, since there aren’t any spatial or temporal limits, this power have no limits and every place of the paramount reality can be always potentially re-shaped by these games.

We focussed our attention on Augmented Reality games and not on Augmented Reality in general even if we started our work seeking the effects of Augmented Reality and not specifically of games. However, in our analysis we always referred to these games starting from a perceptual point of view by analysing how the perception of digital objects is always enclosed within a specific province of meaning. It was the play in perception among these different worlds which produces the hybridisation of the world where we live. We analysed video games, but our analysis is founded on how subjects perceive and not on the fact these Augmented Realities are video games. Thus, we can appreciate the importance of having used a phenomenological approach because our study works in every case where Augmented Reality yields to this shift in perception among different worlds, and it is not restricted to games.^41^

The space around the subjects becomes inevitably filled with characteristics coming from the provinces of meaning of Augmented Realities in general.

## Conclusions

In the first section, we introduced four Augmented Reality games in order to have examples of actual applications. We showed how they aim to intertwine digital elements in our everyday world, and we highlighted how these objects are perceived by the players. Thanks to Husserl’s and Schütz’s analysis, we highlighted how these objects are visualised around the subjects, and how this visualisation is not enough to make them part of the everyday world of the subjects. Even if they are merged with the surroundings thanks to the GPS location and the superimposition of their images on the ones of the world around, they are still related to a finite province of meaning instead of being part of the paramount reality.

In the second part, thanks to the Schütz’s phenomenology, we analysed the interactions between different realities, and we showed how these worlds actually affect each other. Therefore, even if the objects are generated within the finite province of meaning of game, they do have effects on other provinces and on the paramount reality. More precisely, we showed that the actions made by the players within the game cannot be confined to the game because they are performed in space and time which are part of the paramount reality too. Even if their meanings in the game are restricted to a finite province of meaning, they have the chance to affect the other realities through the elements they have in common such as spatial and temporal positions. A “valuable” location in the game always affects the relevance of that location in the paramount reality.

Spatial and temporal positions are important elements on which found these influences among realities, but they are not the only ones. Many other elements can ground the hybridisation of these different worlds. For example, the social relations generated within the game or the power relations which bind players together can be other elements for their hybridisation. Members of a online game could gather in a meeting in the paramount reality in order to know each other in the “real world” or the hierarchies within a game could move people to act in particular ways even in the paramount reality. However, in this work, we just wanted to show the possibility of effects of these games on the everyday world and so we can just introduce these elements without going into details.

We can find these effects related to any province of meanings like with the world generated by films, with books, with games like chess and the play of children. However, in the case of Augmented Reality we do have some differences. We have shown that AR games are not limited in space and time and so we highlighted how they are going to “touch” and influence the world in a much more pervasive way. The values generated within the AR cannot be limited within it because they are always spread all over the globe. Our phenomenological analysis allows us to show the links between these different realities and how they affect each other starting from the experiences of the subjects living in these worlds.

We should not underestimate this contaminations between realities because it is a way to change our world indirectly. For example, this kind of hybridisation between realities could be used in order to change the relevance of places in the paramount reality by someone. A big brand could make agreements with the designers of these games in order to add value to specific places they are interested in like stores. By making a store a valuable place where Pokémon often appear, the importance of that place in the paramount reality changes according to the new value acquired in the game. *McDonald* is one of the sponsors of *Poémon Go* in Japan and they have agreement (Abigal 2016; Mochizuki 2016) in turning every *McDonald* store into a “valuable” place for the game. Thanks to this game-related agreement, they change the relevance of the place in the paramount reality by adding value to those locations in the game for the players. Moreover, as we showed, this hybridisation is not limited to the players because it touches even the ones who do not play in the game. The effects are pervasive and they touch the entire society we live in.

The digital objects of these applications are part of provinces of meaning of the games. Thus the intertwinement between digital and everyday world aimed by Augmented Reality is not achieved yet because these objects are still fictitious and they are not part of the everyday world. However, they do affect the paramount reality where the players and the non-players live their everyday lives. The aim of Augmented Reality is not achieved yet, but it does not mean these objects have no influence on our everyday world at all. Our world is digitally embedded even if digital objects are not here among us. Who design these games have the possibility of shaping our world from within their game.

Maybe the Pokémon do not walk on this Earth hands in hands with us, but they do shape our world.

## Footnotes

1 For example, mobile devices allow subjects to be always connected to digital database where our personal information like schedules are stored.

2 With “Augmented reality” we identify an environment where digital objects are introduced in the everyday world (Milgram 1994; Azuma et al. 2001; Azuma 1997; Furht 2011).

3
http://www.pokemongo.com/en-us/ (Accessed 18 August, 2016).

4 With this kind of term we refer to every application mixing digital and everyday elements in a wide sense (Milgram 1994; Krueger 1983, 1991; Mann and Fung 2002; Lackey and Shumaker 2016).

5 Especially we will refer to the application *Geocaching* by *Groundspeak, Inc.* https://www.geocaching.com/play (Accessed 18 August, 2016).

6
http://www.gamefaqs.com/iphone/982171-ibutterfly (Accessed 18 August, 2016).

7
https://www.ingress.com/ (Accessed 18 August, 2016).

8
http://www.pokemongo.com/en-us/ (Accessed 18 August, 2016).

9 This system was officially used for the first time the 3rd of May, 2000, and, from that moment on, many people joined this community. Thanks to an application for mobile devices, now it is really easy to play the game, and the pool of users is becoming bigger and bigger.

10 We will not use the difference between gamers and players highlighted by Goffman. According to his distinction, the gamers interact even with actions not strictly defined by the rules of the game, while the players represent subjects strictly defined within the mere “rules” of the game (Goffman 1961: 33).

11 We will describe these games in order to provide the readers an idea on how they work and how they merge digital elements and objects in the real world. However, we will follow a phenomenological analysis, and so we will not go into details of case studies.

12 We use the term “real” term as opposed to “digital” and “virtual”. Thus “real objects” are the ones which are not visualised by digital technologies and the “digital objects” are the ones generated by Augmented Reality and visualised through computer devices.

13 Objects were found on the top of mountains, caves or even under the sea (https://www.geocaching.com/blog/2015/08/the-wild-world-of-underwater-geocaches/) (Accessed 18 August, 2016).

14 In *Harry Potter*, “muggles” are the human beings who do not have any particular magic power and they cannot see magical objects or magic in general.

15 In this application, there is a map showing places where to find digital butterflies. However, the map is not used as in the previous case because it does not point to specific objects or butterflies directly, but to places where subjects can find them. In the case of *Geocahing*, the map points to objects. Thus, we can ignore this aspect of the game because it is not similar to the other applications. Also in the case of *Ingress* and *Pokémon Go*, we have a map pointing to objects and not places.

16 Sometimes the entire film is developed by digital technologies and there are no real characters or objects in it. See, for, example, the films by *Pixar*.

17 Husserl uses the term “positional act” in order to identify the action of the subjects when they believe in the existence of an object. He clearly states that in the case of theatre or paintings, subjects suspend this act because they live within that world (Husserl 1980; Brough 1992).

18 Husserl refers to three different images the subjects deal with when they are in front of a painting (1980: 18f.): physical image [*physische Bild*], the image-object [*das Bildobjekt*], and the image-subject [*das Bildsubjekt*] (Uzelac 1998).

19 “Husserl calls this internal relation ‘seen-in’ or—more precisely—‘look into’ [*Hineinsehen*] [...], by which he not only means that one sees something in the picture things, such as photo paper, but also that we ‘feel’ ourselves in the picture object such as a photo”(Lotz 2007: 174). See also Casebier (1991), de Warren (2010) and Liberati (2013).

20 The term “immersion” is used not only to identify the feeling of being in it, but it is used to identify also other aspects like the players’ commitment to the story (Linderoth 2012; McMahan 2003).

21 They do have effects on the outside world like in the case of a comedy which leaves the spectators happy even after the end of the film.

22 In order to have an analysis more related to game studies on the perception of an entire world within a “door,” see Aarseth (2005).

23 We analysed the case of a film, but we can easily adopt this analysis with other cases like with Virtual Realities. In the case of Virtual Reality, we have the same kind of immersion. A subject, thanks to devices, can “access” a different digital world where objects are presented as perceptually real, but which are not existent in the world “outside” (Warren 2014; Liberati 2013).

24 We could use the analysis developed by game studies on the “magic circle” because it directly focusses on this topic (Stenros 2012; Zimmerman 2012; Salen and Zimmerman 2003). However, we will tackle the topic from a phenomenological analysis in order to analyse their emergence from a perceptual point of view. In this way, even if this analysis concerns games, we do not limit our analysis to them only, but potentially it can our study can be applied every other praxes which generates new objects in the surroundings. For a connection between Huizinga’s analysis and phenomenological one, see Rowlands (2016) and Istrate (2011).

25 “Among the multiple realities there is one that presents itself as the reality *par excellence*. This is the reality of everyday life. Its privileged position entitles it to the designation of paramount reality. The tension of consciousness is highest in everyday life, that is, the latter imposes itself upon consciousness in the most massive, urgent and intense manner” (Berger and Luckmann 1991: 35).

26 In literature (Schutz 2013) we have the same entrance to another world defined by the text of a book (Sebald 2011).

27 For example, the monsters presented by a horror film do not exist in the paramount reality and their existence is enclosed in the finite province generated by the film. On a phenomenological analysis on films see Sobchack (1992) and Baudry and Williams (1974). More in details, Baudry’s theory can be applied to Augmented Reality (Rhodes 2014), and it is possible to find analyses on cinema and Augmented Reality such as (Jackman 2015).

28 On the relations between Goffman and Schütz, see Psathas (2014).

29 As we have previously highlighted, it is possible to refer to other authors in order to study the game of chess within a different framework, such as in the case of Huizinga’s analysis (1938).

30 In order to understand how augmented reality could produce digital objects which are part of the paramount reality, see Liberati (2015a).

31 Even in this case, it is possible to refer to the analysis made by game studies like in the case of Juul (2005) where the video-game are seen as something between real and fiction. However, we will just use references related to phenomenological tradition.

32 This is valid also for the other games we analysed, but we are taking into consideration only *Pokémon Go* to make the text as fluid as possible.

33 This selection can be active or passive. The subjects can actively decide to immerse themselves in a finite province of meaning as when they enter in cinema or when they read a book. However, they can fall within a reality without having any power on it as in the case of dreams where subjects do not decide to enter them and they have no power on them.

34 According to Schütz, subjects can change the world where they live by experiencing shocks (Schütz 1962).

35 Schütz calls this kind of time “cosmic time” (Schutz and Wagner 1970).

36 In infinite games, there is no clear winner because the sole goal of these games is to continue playing (Carse 1986: 3) and not to defeat an enemy. An example of infinite game is *Dungeons and Dragons* (Cogburn and Silcox 2012). As the film *Mazes and Mosnters* directed by Steven Hilliard Stern suggests, people could immerse themselves for their entire life because there is no end in the game.

37 In the case of *Ingress*, we have two factions trying to defeat the other. However, the game is structured in order to never make one of this faction lose. As in the game *Dungeouns and Dragons* which clearly has monsters to be defeated, the game never ends because the monsters are merely an “excuse” to keep the game going on.

38 These Augmented Reality games have boundaries dictated by the infrastructure they depend on. Without a system which provides internet access and a precise GPS location, they simply cannot work. However, these limits are related to the infrastructure which supports the game and not to the game in itself. Potentially, these games can be played everywhere and at any time.

39 Using Schützian terminology these subjects “grow old” together (Schütz 1962; Zahavi 2014).

40 Unfortunately, there are many examples of accident produced by intertwinements between the action of players and the action of non-players. For example, the fact that a person is driving a car while playing to *Pokémon Go* is quite significant for the subject who do not play *Pokémon Go* as well. If the driver/player is so captured by the finite province of meaning generated by *Pokémon Go*, the driver will not be able to shift from this fictional world to the paramount reality quickly enough to stop the car in case of a pedestrian crossing the street or in case the street has a red traffic light (http://www.usatoday.com/story/news/nation-now/2016/07/20/driver-slams-into-baltimore-cop-car-while-playing-pokemon-go-accident/87333892/) (Accessed 18 August, 2016). This kind of accident is so usual that *Niantic* had to expressively warn the players at the beginning of the play not to drive (Mogg 2016).

41 We can provide few examples on how our analysis can be applied to AR applications which are not games. Our analysis can be applied to augmented realities designed for tourism and museums where digital objects are visualised in order to let the user enjoy the history related to a place or to an ancient object. These applications are not games, but they still fall within our study (Neuburger and Egger 2018). We can also apply our analysis to applications designed for education where 3D objects are generated by the augmented reality. For example, in medicine, the human body can be visualised as a 3D object thanks to AR. These kinds of Augmented Reality have the same elements of *Pokémon Go* since they provide digital objects visualised through a device and which are perceived by the mere users of that specific device (Zhu et al. 2014; Chen et al. 2017; Barsom et al. 2016). Therefore, our analysis shows how these digital objects can be treated as the Pokémon since they are perceived in the same way by the users.

## References

[CR1] Aarseth, E. (2005). Doors and perception: Fiction vs simulation in games. In *Digital arts and culture proceedings*, IT University of Copenhagen. http://www.luisfilipeteixeira.com/fileManager/file/fiction_Aarseth_jan2006.pdf.

[CR2] Abigal, A. (2016). McDonald’s just became the first major company to partner with Pokémon Go. *TIME*. http://time.com/4417311/mcdonalds-pokemon-go-partership/.

[CR3] Aceti, L., Rinehart, R., Sahin, O., et al. (2013). Not here not there. *Leonardo Electronic Almanac, 19*(2). http://www.leoalmanac.org/vol19-no2-not-here-not-there-part-2/. Accessed 29 Nov 2013.

[CR4] Ashcraft, B. (2016). *Japanese Pokémon Go players fall for Mewtwo Prank*. http://kotaku.com/a-perfect-place-for-a-pokemon-go-rumor-1784243688.

[CR5] Ayaß R (2016). Life-world, sub-worlds, after-worlds: The various ‘realnesses’ of multiple realities. Human Studies.

[CR6] Azuma RT (1997). A survey of augmented reality. Presence: Teleoperators and Virtual Environments.

[CR7] Azuma RT, Baillot Y, Behringer R, Feiner S, Julier S, MacIntyre B (2001). Recent advances in augmented reality. IEEE Computer Graphics and Applications.

[CR8] Barber MD (2015). Resistance to pragmatic tendencies in the world of working in the religious finite province of meaning. Human Studies.

[CR9] Barsom EZ, Graafland M, Schijven MP (2016). Systematic review on the effectiveness of augmented reality applications in medical training. Surgical Endoscopy.

[CR10] Baudry JL, Williams A (1974). Ideological effects of the basic cinematographic apparatus. Film Quarterly.

[CR11] Berger PL, Luckmann T (1991). The social construction of reality: A treatise in the sociology of knowledge.

[CR12] Billinghurst M, Clark A, Lee G (2015). A survey of augmented reality. Foundations and Trends® in Human–Computer Interaction.

[CR13] Bischur D, Staudigl M, Berguno G (2014). Scientific practice and the world of working. Beyond Schutz’s Wirkwelt. Schutzian phenomenology and hermeneutic traditions. Contributions to phenomenology.

[CR14] Bolter, J., Engberg, M., & MacIntyre, B. (2013). Media studies, mobile augmented reality, and interaction design. *Interactions*, *20*(1), 36–45. http://interactions.acm.org/archive/view/january-february-2013/media-studies-mobile-augmented-reality-and-interaction-design.

[CR15] Brough J (1992). Some Husserlian comments on depiction and art. The American Catholic Philosopical Quarterly.

[CR16] Carse, J. P. (1986). *Finite and infinite games: A vision of life as play and possibility*. Free Press. http://libgen.io/ads.php?md5=0C63EC966F4150801E31D6C55E8195F6.

[CR17] Casebier A (1991). Film and phenomenology: Toward a realist theory of cinematic representation.

[CR18] Caudell TP (1995). Introduction to augmented and virtual reality. Proceedings of SPIE.

[CR19] Caudell, T. P., & Mizell, D. W. (2002). Augmented reality: an application of heads-up display technology to manual manufacturing processes. In *Proceedings of the twenty-fifth Hawaii international conference on system sciences* (Vol. 2, pp. 659–669). 10.1109/hicss.1992.183317.

[CR20] Chen, L., Day, T., Tang, W., & John, N. W. (2017). Recent developments and future challenges in medical mixed reality. arXiv:1708.01225

[CR21] Chojnacki, M. (2012). “Secularization” or plurality of meaning structures? A. Schutz’s concept of a finite province of meaning and the question of religious rationality an ambitious question profiting from a major ambiguity. *Open Journal of Philosophy*, *2*(2), 92–99. 10.4236/ojpp.2012.22014. http://www.SciRP.org/journal/ojpp www.SciRP.org/journal/ojpp.

[CR22] Cogburn J, Silcox M (2012). Dungeons and dragons and philosophy: Raiding the temple of wisdom.

[CR73] De Warren N, Ierna C, Jacobs H, Mattens F (2010). Tamino’s eyes, Pamina’s gaze: Husserl’s phenomenology of image-consciousness refashioned. Philosophy, phenomenology, sciences: Essays in commemoration of Edmund Husserl, phaenomenologica.

[CR74] de Warren N (2014). Towards a phenomenological analysis of virtual fictions. Metodo International Studies in Phenomenology and Philosophy.

[CR23] Ducharme LJ, Fine GA, Bogue R, Spariosu MI (1994). No escaping obligation Erving Goffman on the demands and constraints of play. The play of the self.

[CR24] Engberg M, Bolter JD (2014). Cultural expression in augmented and mixed reality. Convergence: The International Journal of Research into New Media Technologies.

[CR25] Fine GA (2002). Shared fantasy: Role playing games as social worlds.

[CR26] Foster, C. (2012). *Augmented reality game gets player busted: The first of many?* http://readwrite.com/2012/12/11/augmented-reality-game-gets-player-arrested-the-first-of-many/.

[CR27] Furht, B. (Ed.). (2011). *Handbook of augmented reality*. Springer. http://dblp.uni-trier.de/db/books/daglib/0027797.html.

[CR28] Geroimenko V (2014). Augmented reality art: From an emerging technology to a novel creative medium. Springer series on cultural computing.

[CR29] Goffman E (1961). Encounters: Two studies in the sociology of interaction.

[CR30] Goffman E (1974). Frame analysis: An essay on the organization of experience.

[CR31] Huizinga J (1938). Homo ludens.

[CR32] Husserl, E. (1980). *Phantasie, Bildbewußtsein, Erinnerung, Husserliana* (Vol. XXIII). Springer.

[CR33] Ihde D (1990). Technology and the lifeworld: From garden to earth.

[CR34] Istrate AM (2011). From pathological to professional: Gambling stories. Journal of Comparative Research in Anthropology and Sociology.

[CR35] Jackman AH (2015). 3-D cinema: Immersive media technology. GeoJournal.

[CR36] Juul J (2005). Half-real: Video games between real rules and fictional worlds.

[CR37] Krueger MW (1983). Artificial reality.

[CR38] Krueger MW (1991). Artificial reality II.

[CR39] Lackey S, Shumaker R (2016). Virtual, augmented and mixed reality.

[CR40] Liberati, N. (2013). Improving the embodiment relations by means of phenomenological analysis on the “reality” of ARs. In *IEEE international symposium on mixed and augmented reality arts, media, and humanities (ISMAR-AMH)* (pp. 13–17). 10.1109/ISMAR-AMH.2012.6483983.

[CR41] Liberati, N. (2015a). Augmented “Ouch”. How to create intersubjective augmented objects into which we can bump. In *IEEE international symposium on mixed and augmented reality-media, art, social science, humanities and design* (pp. 21–26). IEEE. 10.1109/ISMAR-MASHD.2015.14. http://ieeexplore.ieee.org/lpdocs/epic03/wrapper.htm?arnumber=7350730.

[CR42] Liberati N, Cheok AD (2015). “Digital materiality” and augmented reality. Hyperconnectivity and the future of internet communication.

[CR43] Liberati N (2016). Technology, phenomenology and the everyday world: A phenomenological analysis on how technologies mould our world. Human Studies.

[CR44] Linderoth J (2012). The effort of being in a fictional world: Upkeyings and laminated frames in MMORPGs. Symbolic Interaction.

[CR45] Lotz C (2007). Depiction and plastic perception. A critique of Husserl’s theory of picture consciousness. Continental Philosophy Review.

[CR46] Mann S, Fung J (2002). EyeTap devices for augmented, deliberately diminished, or otherwise altered visual perception of rigid planar patches of real-world scenes. PresenceTeleoperators and Virtual Environments.

[CR47] Manovich L (2006). The poetics of augmented space. Visual Communication.

[CR48] McDuffie MF, Crowell SG (1995). Art as an enclave of meaning. The prism of the self. Contributions to phenomenology (In cooperation with the center for advanced research in phenomenology).

[CR49] McMahan A, Wolf MJP, Perron B (2003). Immersion, engagement, and presence: A method for analyzing 3-D video games. The video game theory reader.

[CR50] Milgram P (1994). Augmented reality: A class of displays on the reality-virtuallity continuum. SPIE Telemanipulator and Telepresence Technologies.

[CR51] Mochizuki, T. (2016). McDonald’s unit to sponsor ‘Pokémon Go’ in Japan. *The Wall Street Journal*. http://www.wsj.com/articles/mcdonalds-unit-to-sponsor-pokemon-go-in-japan-1468936459.

[CR52] Mogg, T. (2016). *Pokémon Go update wants you to confirm you’re not driving*. http://www.digitaltrends.com/gaming/pokemon-go-driving-message/.

[CR53] Neuburger L, Egger R, Jung T, Tom Dieck M (2018). Augmented reality: Providing a different dimension for museum visitors. Augmented reality: Providing a different dimension for museum visitors.

[CR54] Psathas G, Embree L (1998). On multiple realities and the world of film. Alfred Schutz’s “Sociological Aspect of Literature”. Contributions to phenomenology (In cooperation with the center for advanced research in phenomenology).

[CR55] Psathas G, Staudigl M, Berguno G (2014). Goffman and Schutz on multiple realities. Schutzian phenomenology and hermeneutic traditions. Contributions to phenomenology.

[CR56] Rhodes G, Geroimenko V (2014). Augmented reality in art: Aesthetics and material for expression. Augmented reality art.

[CR57] Rowlands, T. (2016). *Video game worlds: Working at play in the culture of EverQuest*—Timothy Rowlands, Google Books.

[CR58] Salen, K., & Zimmerman, E. (2003). *Rules of play: Game design fundamentals*. MIT Press. https://www.google.nl/search?q=zimmerman+Rules+of+Play.&ie=utf-8&oe=utf-8&client=firefox-b&gfe_rd=cr&ei=oHdbWeDDF6_VXrOTnoAC.

[CR59] Schraffenberger, H., & Manovich, L. (2013). *Interview AR[t] Magazine*. http://www.creativecode.org/wp-content/uploads/2015/01/ARt3_ForwardedMessage.pdf.

[CR60] Schraffenberger, H., & van der Heide, E. (2014). The real in augmented reality. In M. Carvalhais, & M. Verdicchio (Eds.), * Proceedings of the second conference on computation, communication, aesthetics and X, xCoAx* (pp. 64–74).

[CR61] Schütz, A. (1962). *Collected papers: The problem of social reality, phaenomenologica* (Vol. 11). Martinus Nijhoff.

[CR62] Schutz A, Barber M (2013). Meaning structures of literary art forms. Collected papers VI. Literary reality and relationships. Phaenomenologica (Series founded by H.L. van Breda and published under the auspices of the Husserl-Archives).

[CR63] Schutz A, Wagner HR (1970). On phenomenology and social relations: Selected writings.

[CR64] Sebald G (2011). Crossing the finite provinces of meaning. Experience and metaphor. Human Studies.

[CR65] Shilkrot, R., Montfort, N., & Maes, P. (2014). nARratives of augmented worlds. In *2014 IEEE international symposium on mixed and augmented reality-media, art, social science, humanities and design (IMSAR-MASH’D)* (pp. 35–42). IEEE. 10.1109/ISMAR-AMH.2014.6935436.

[CR66] Sobchack VC (1992). The address of the eye: A phenomenology of film experience.

[CR67] Steets S (2014). Multiple realities and religion: A sociological approach. Society.

[CR68] Stenros, J. (2012). In defence of a magic circle: The social and mental boundaries of play. In *Proceedings of DiGRA Nordic 2012 conference: Local and global-games in culture and society* (pp. 1–19). http://www.digra.org/wp-content/uploads/digital-library/12168.43543.pdf.

[CR69] Tuckett J (2015). Extending the dimensions of the social world through game-worlds. Gamevironments.

[CR70] Uzelac M (1998). Art and phenomenology in Edmund Husserl. Axiomathes.

[CR71] Verbeek PP (2005). What things do. Philosophical reflections on technology, agency, and design.

[CR72] Waltemathe M, Grieve GP, Heidi AC (2014). Bridging multiple realities: Religion, play and Alfred Schutz’s theory of the life-world. Playing with religion in digital games.

[CR75] Wassom B, Bishop A (2014). Augmented reality law, privacy, and ethics: Law, society, and emerging AR technologies.

[CR76] Wellner, G. (2013). No longer a phone: The cellphone as an enabler of augmented reality. *Transfers, 3*(2), 70–88. http://www.berghahnjournals.com/view/journals/transfers/3/2/trans030205.xml?pdfVersion=true.

[CR77] Wellner GP (2015). A postphenomenological inquiry of cell phones: Genealogies, meanings, and becoming.

[CR78] Zahavi D (2014). Self and other: Exploring subjectivity, empathy, and shame.

[CR79] Zhu, E., Hadadgar, A., Masiello, I., & Zary, N. (2014). Augmented reality in healthcare education: an integrative review. *PeerJ*, *2*, e469. 10.7717/peerj.469. http://www.ncbi.nlm.nih.gov/pubmed/25071992http://www.pubmedcentral.nih.gov/articlerender.fcgi?artid=PMC4103088. PMC4103088 25071992

[CR80] Zimmerman, E. (2012).* Jerked around by the magic circle—clearing the air ten years later*. http://www.gamasutra.com/view/feature/135063/jerked_around_by_the_magic_circle_.php.

